# Effect of evening primrose oil on postoperative pain after appendectomy: A double-blind, randomized, clinical trial

**DOI:** 10.37796/2211-8039.1002

**Published:** 2020-03-28

**Authors:** Manijeh Yousefi Moghadam, Mohammad Nemat-Shahi, Davood Soroosh, Mahbobeh Nemat-Shahi, Atefeh Asadi

**Affiliations:** a Department of Anesthesiology, Faculty of Medicine, Sabzevar University of Medical Sciences, Sabzevar, Iran; b Faculty of Medicine, Sabzevar University of Medical Sciences Sabzevar, Iran; c Department of Community Medicine, Faculty of Medicine, Sabzevar University of Medical Sciences Sabzevar, Iran; d Deputy of Health, Sabzevar University of Medical Sciences Sabzevar, Iran

**Keywords:** appendectomy, evening primrose, *Oenothera biennis*, pain, postoperative

## Abstract

**Background and objective:**

Despite advances in surgical techniques and pharmacology, postoperative pain remains a common problem after appendectomy, and its management continues to be suboptimal. The aim of this study was to evaluate the effect of evening primrose oil on the reduction of postoperative pain after appendectomy.

**Materials and methods:**

In a double-blind, randomized, clinical trial, a total of 80 adults patients with acute appendicitis who were undergoing appendectomy at the Shahid Beheshti Emdad Hospital in Sabzevar, were included. Patients were randomly allocated into two equally sized groups (n = 40). In postoperative period and after recovering from the anesthesia, each of the intervention and control groups received one evening primrose (1000 mg) or placebo capsules every 30 min for 3 times, respectively. All patients in both groups were asked to rate the intensity of their pain on a 0–10 point Visual Analogue Scale (VAS) and also McGill pain questionnaire, before and 1 h after the last administration of the drug, postoperatively.

**Results:**

In patients who received evening primrose, both VAS and McGill pain intensity scores significantly decreased after intervention, when compared prior to initiation of the intervention (p < 0.0001). While in the control group, changes of pain intensity scores were not significantly different before and after the intervention (p > 0.05).

**Conclusion:**

It seems that oral evening primrose can be used as a simple and safe potential adjunctive treatment for postoperative pain control after appendectomy.

## 1. Introduction

Postoperative pain is one of the most frequent complaints after surgery. Most people experience pain in postoperative periods, for various reasons. It has been shown that despite advances in surgical techniques and pharmacology, up to 75% of patients in surgical wards experience moderate to severe postoperative pain [1–4]. Inappropriate postoperative pain control is associated with patients' dissatisfaction, increased hospital stay and costs, increased morbidity and risk of developing chronic pain [5–11]. Todays, opioids such as meperidine hydrochloride, morphine sulfate and non-steroidal anti-inflammatory drugs (NSAID) are among most commonly used medication for postoperative pain management [12–16]. Using these medication is potentially associated with various side effects such as nausea, vomiting, respiratory depression, hypotension, sedation and gastrointestinal bleeding, which can consequently lead to insufficient postoperative pain treatment [1,14–16]. Inflammation has been proposed as one of the possible mechanisms of postoperative pain. Therefore, using pharmacologic and medicinal plants with anti-inflammatory activities are reasonable for managing postoperative pain [1,17,18].

Medicinal plants have been commonplace since ancient times, and today they are commonly used in various forms, including herbal plants or their extracts throughout the world [19–21]. Evening primrose is proven to anti-inflammatory effects; increased omega-6 fatty acids; improved vasodilator synthesis; correction of nerve blood flow and nerve conduction velocity defects in diabetic patients. Gamma-linolenic acid (GLA) is one of the essential fatty acids that the body cannot produce and like vitamins, should reach the body through food and supplements. This fatty acid, which is present in evening primrose, increases the production of prostaglandin E1, which has anti-inflammatory effects [22–24]. Since 1970, 1000 tons of this oil has been used daily in different countries, so far no complaints have been reported about its side effects. Also, many clinical trials have shown the beneficial effects of EPO as a source of GLA in cases such as diabetic neuropathy, hypertension, breast pain, premenstrual syndrome, osteoporosis, dementia and hysteroscopy [19,22,25,26].

To the best of our knowledge, despite the promising anti-inflammatory and analgesic effect of evening primrose oil and potential benefit of using evening primrose oil as an adjuvant for postoperative pain management, no published study evaluated its efficacy on postoperative pain.

Therefore, the aim of this study was to evaluate the effect of evening primrose on postoperative pain after appendectomy.

## 2. Methods

In a double-blind randomized, clinical trial, a total of 80 patients who were undergoing appendectomy and hospitalized in Emdad Shahid Beheshti Hospital in Sabzevar, Iran, were evaluated from November 2016 to May 2017. After obtaining approval from the institutional ethics committee and written informed consent from the patients, those who meet the inclusion criteria were randomly allocated into two intervention and control groups (n = 40). Inclusion criteria were Class I or II of American Society of Anesthesiologists, aged 25–75 years and weight between 40 and 120 kg. Exclusion criteria were pregnancy or lactation, previous history of surgery in the last three years; known systemic or mental psychiatric disorders such as epilepsy or schizophrenia; drugs abuser and receiving more than 2 μg/kg of fentanyl during anesthesia Also, patients treated with steroids or non-steroidal anti-inflammatory drugs were excluded from the study.

After surgery, in the presence of consciousness and gag reflex (mean time of 20 min), patients in intervention group received an evening primrose capsule (Natural Life™, Australia) every 30 min for three times, and the control group received a placebo capsule every 30 min for three times as well. One hour after last administration of the drug, patients' pain intensity were evaluated using the visual analog scale (VAS) and McGill Pain Questionnaire. All patients in both groups received Midazolam (0.02 mg/kg) for premedication, fentanyl (2 μg/kg), sodium thiopental (55 μg/kg) and 1.5 μg/kg Aesculin and 0.5 μg/kg Atracurium for induction of anesthesia. In order to maintain anesthesia, 50% Nitrous oxide, 50% oxygen and isoflurane at 1.2 MAC concentrations were given to each patient. In recovery, 1 μg/kg Fentanyl was used to relieve pain. Therefore, all patients in both groups were the same for the drugs received during and after surgery. The patients and the researcher did not know the contents of the capsules (double-blind). The evening primrose capsule contained 1000 mg and placebo capsules contained 1000 mg wheat flour. Before administration of the drugs, an anesthesia nurse who was not aware of the patients' group, evaluated patients' pain intensity and then prescribed capsules as A and B (every 30 min for 3 times) to each group. One hour after the last administration of the drug, the nurse completed the questionnaire and a checklist again. All patients in intervention group were followed for any potential adverse effects of evening primrose oil such as headache, bloating, diarrhea or abdominal pain.

The data were analyzed using SPSS 20 software and Student T-test and Chi-square software.

This study was registered in the Iranian Registry of Clinical Trials Database (IRCT2017092533202N3).

## 3. Results

**Fig. 1:**
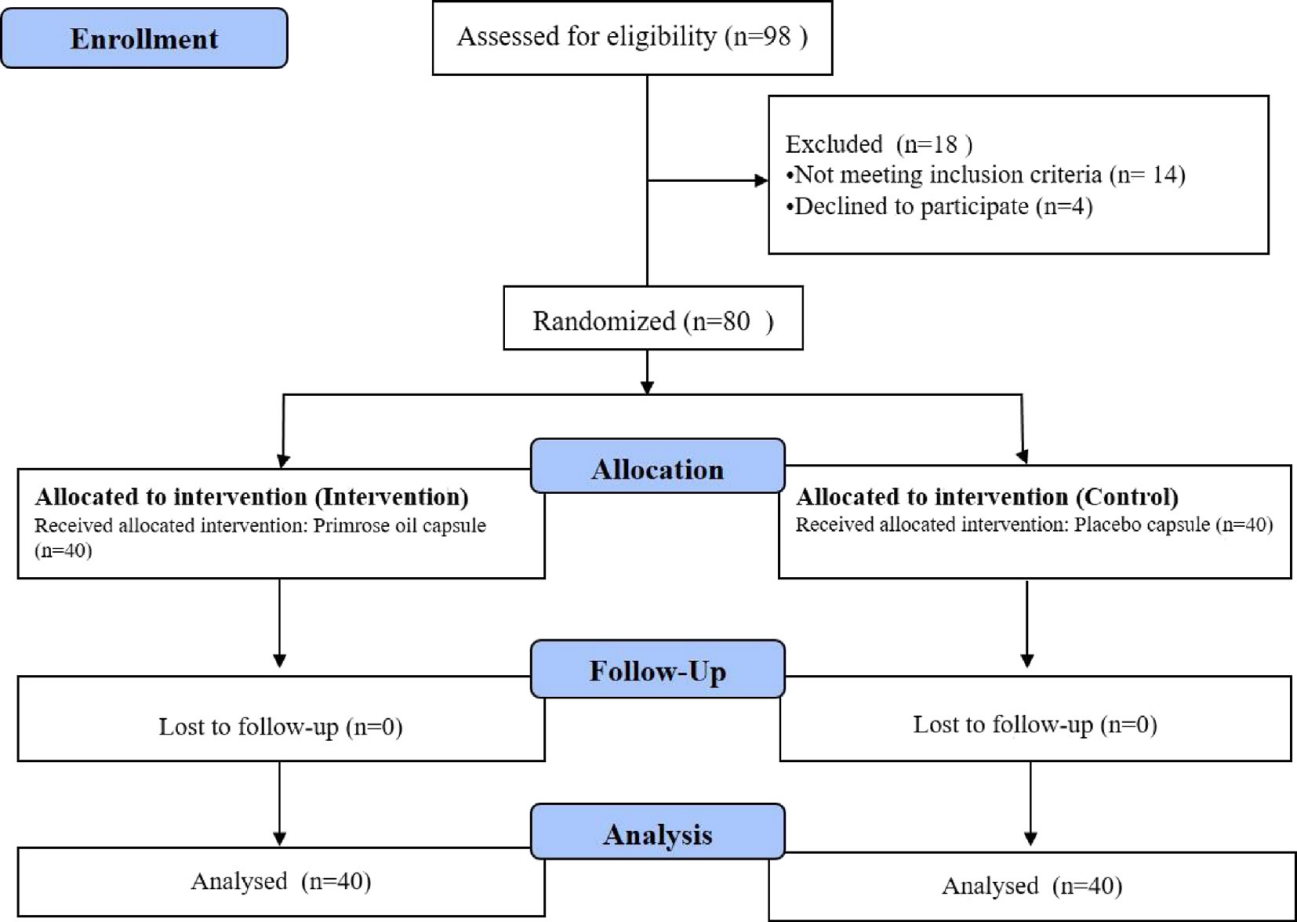
Flow chart of the study.

A total of 98 patients were screened during the study period. Of these, 14 patients did not meet the inclusion criteria and 4 patients declined to participate in the study (Fig. 1).

The two groups were similar and no significant difference was observed in terms of demographic characteristics and average age. In this study, the mean age of patients was 34 ± 2 years (minimum 21 and maximum 45 years). In terms of gender, 82.5% of the subjects were male and 17.5% were female (Table 1).

The results of the study showed that the two groups were not statistically significantly different in terms of pain intensity prior to initiation of the intervention. In the postoperative period, the pain intensity (using both VAS and McGill questionnaires) significantly decreased in patients who received evening primrose oil when compared prior to initiation of the intervention; while in the control group the changes of pain intensity after surgery was not statistically significant (Table 2).

No evening primrose oil-related adverse effects were observed in the study.

## 4. Discussion

To the best of our knowledge, this is the first study to evaluate the effects of evening primrose oil in managing acute postoperative pain. The results of this study showed that using evening primrose oil significantly reduces postoperative pain in patients undergoing appendectomy. It has been previously confirmed that evening primrose can be used to treat many diseases with chronic inflammation [22]. For many years, using evening primrose, as a supplement, has been recommended for treatment of mastalgia [25,27,28]. In a study by Jaafarnejad et al. has been shown that daily using 2000 mg of evening primrose can reduce the duration of breast pain [29]. Additionally, it has been indicated that 6- month usage of 3000 mg of evening primrose oil significantly decreases the intensity of premenstrual cyclical breast discomfort [30]. Another study in women showed that evening primrose oil therapy can significantly decrease and improve premenstrual syndrome symptom severity [31]. Also, Jäntti et al. indicated that twice daily administration of 10 ml evening primrose oil for 12 weeks significantly increases plasma prostaglandins concentration in patients with rheumatoid arthritis, however no significant clinical improvement has been reported [32]. Another study by Brzeski et al. showed that using evening primrose oil 6 g/day leads to a mild improvement in patients with rheumatoid arthritis [33]. A review study by Cameron et al. [34] showed that oils containing GLA (Primrose, borage, or Black Grape Seed Oil) can relieve the symptoms of patients with rheumatoid arthritis. Saki, in a study that investigated the effect of evening primrose oil on depression, stated that although the mean score of depression and the patients' performance had been significantly reduced at the beginning of the study, weeks 4, 8, and 12 in both groups (evening primrose oil or nortriptyline therapy), in patients who received evening primrose oil, the decrease was more significant [35]. In a study, which has been conducted on patients with multiple sclerosis, it has been shown that using 1 g of evening primrose oil every 12 h for 3 months can significantly decrease pain and fatigue and also increase their life satisfaction, cognitive function and vitality [36]. SafaaHussain et al. evaluated the effect of evening primrose oil in type 2 diabetic patients. The result of this study showed that administration of three-month evening primrose oil, in combination with conventional treatment, can significantly decrease the level of blood inflammatory markers, improve therapeutic benefits and decrease diabetes related complications [37]. Our patients received 3000 mg of oral evening primrose oil totally during the study period. Some sources consider right daily intake of evening primrose oil about 8 g for adults and 4 g for children [22,23,25,38,39]. In terms of side effects and safety of evening primrose in humans, although it is usually well tolerated and no significant side effects have been documented in the medical literature, it has been recommended that using evening primrose oil during pregnancy should be avoided [14,40].

## 5. Conclusion

According to the results of this study, it seems that oral evening primrose oil can be used as a simple and safe potential adjunctive treatment for postoperative pain control after appendectomy. However, further well designed study with larger sample size are warranted to evaluate and determine the efficacy and optimal dose of evening primrose oil for managing acute postoperative pain.

## Figures and Tables

**Table 1:** Demographic characteristics of patients in two groups.

Variables	Intervention group	Control group	*P*-value
Gender Male; n (%)	31 (77.5)	35 (87.5)	0.34
Female; n (%)	9 (22.5)	5 (12.5)	
Age (mean ± SD)	33.72 ± 2.89	35.65 ± 2.32	0.19
BMI	21.8 ± 1.7	22.31 ± 1.2	0.27

BMI= Body Mass Index.

**Table 2:** Comparison of pain in the two groups before and after the intervention.

Variables		Intervention Group (Evening primrose oil)	Control Group (Placebo)	*P*- value
Pain intensity before the intervention	VAS	7/78 ± 0/7	8/13 ± 1/2	0.63
	McGill	75/60 ± 5/90	71/55 ± 3/00	0.72
Pain intensity after the intervention	VAS	3/88 ± 0/8	7/00 ± 8/7	<0.0001
	McGill	30/13 ± 4/45	68/03 ± 5/67	<0.0001
